# Cell response to sterilized electrospun poly(ɛ‐caprolactone) scaffolds to aid tendon regeneration *in vivo*

**DOI:** 10.1002/jbm.a.35911

**Published:** 2016-10-18

**Authors:** Prajwal Bhaskar, Lucy A. Bosworth, Richard Wong, Marie A. O'brien, Haydn Kriel, Eugene Smit, Duncan A. McGrouther, Jason K. Wong, Sarah H. Cartmell

**Affiliations:** ^1^ School of MaterialsThe University of ManchesterOxford RoadManchesterM13 9PLUnited Kingdom; ^2^ Institute of Inflammation and Repair, The University of ManchesterOxford RoadManchesterM13 9PLUnited Kingdom; ^3^ The Stellenbosch Nanofiber Company LtdCape TownSouth Africa; ^4^ Department of Chemistry and Polymer ScienceStellenbosch UniversityStellenboschSouth Africa

**Keywords:** tendon repair, electrospinning polycaprolactone, sterilization, gamma irradiation, ethanol

## Abstract

The functional replacement of tendon represents an unmet clinical need in situations of tendon rupture, tendon grafting, and complex tendon reconstruction, as usually there is a finite source of healthy tendon to use as donors. The microfibrous architecture of tendon is critical to the function of tendon. This study investigates the use of electrospun poly(ɛ‐caprolactone) scaffolds as potential biomaterial substitutes for tendon grafts. We assessed the performance of two electrospinning manufacturers (small‐ and large‐scale) and the effect of two sterilization techniques—gamma irradiation and ethanol submersion—on cell response to these electrospun scaffolds after their implantation into a murine tendon model. Cell infiltration and proliferation analyses were undertaken to determine the effect on cell response within the implant over a 6‐week period. Immunohistochemical analysis was performed to characterize inflammatory response and healing characteristics (proliferation, collagen deposition, myofibroblast activity, and apoptosis). Neither the sterilization techniques nor the manufacturer was observed to significantly affect the cell response to the scaffold. At each time point, cell response was similar to the autograft control. This suggests that ethanol submersion can be used for research purposes and that the scaffold can be easily reproduced by a large‐scale manufacturer. These results further imply that this electrospun scaffold may provide an alternative to autograft, thus eliminating the need for sourcing healthy tendon tissue from a secondary site. © 2016 Wiley Periodicals, Inc. J Biomed Mater Res Part A: 105A: 389–397, 2017.

## INTRODUCTION

The effectiveness of autografts in the treatment of major tendon injuries is limited by the relative paucity of donor sites and tissue. Although autografts provide an excellent method of replacement, for large or neglected tendon injuries, supplementation with a tendon‐like material is often desirable.^1^, ^2^, ^3^, ^4^, ^5^ There is an increasing availability of human allograft tendon or xenografts in this area but their availability is limited and are not without risks of disease transmission or immunological reaction.^6^ The decellularization process of these tissues continues to advance; however, studies have shown that recellularization is often suboptimal and tendons appear weaker.^7^ With injury rates rising,^8^, ^9^ there remains a clear clinical need for tendon therapies that regenerate or augment tissue to fully restore functionality.

**Figure 1:**
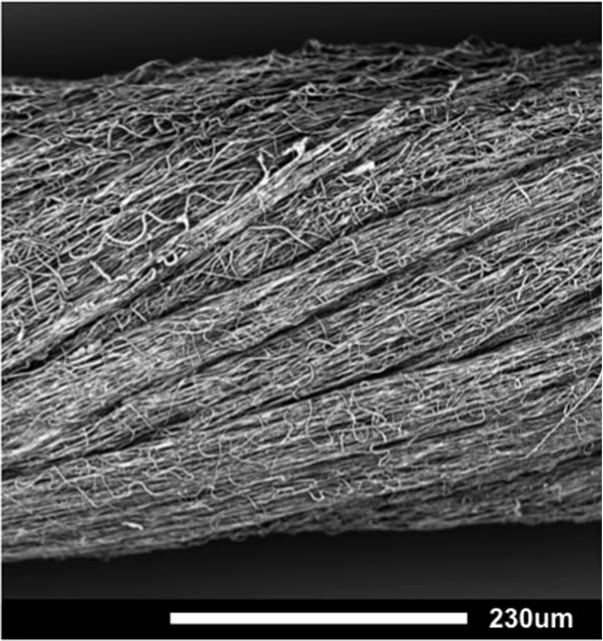
Representative scanning electron microscope (SEM) image of electrospun poly(ε‐caprolactone) (PCL) scaffold. Overall alignment of PCL nanofibers is visible within the macrostructure of the twisted yarn. Scale bar = 230 μm; magnification 580×.

We have researched and developed an electrospun poly(ɛ‐caprolactone) (PCL) scaffold as a novel device intended to aid in tendon regeneration following the loss of tendon tissue.^2^ A long‐term surgically implanted device, in the form of a scaffold, would aim to support the severed ends of the tendon in the short term by imitating the nanofibrous architecture of healthy tendon, as well as facilitating cell recruitment and collagen deposition into functioning tendon tissue over time. The scaffold encompasses an electrospun PCL fiber network that has been fabricated to form a yarn, which can be woven to create scalable structures appropriate for tendons of different size to fit with clinical requirements (Fig. 1).

Before being approved for use in the human body, the PCL scaffold must be demonstrably sterile. As stated by Rogers, sterility can be described as “the complete removal or destruction of viable organisms”.^10^ It is a critical requirement of all medical products and must be considered during the product development stage. A nonsterile medical device used on a patient could cause an elevated inflammatory response, infection, and could lead to implant failure and even patient mortality.^10^

A sterilization process may have a degenerative or a destructive effect on a product, creating a need to validate its functionality poststerilization. Common methods of medical product sterilization include ionizing radiation and, in research laboratory settings (i.e., for nonterminal sterilization), alcohol sterilization.

Ionizing radiation generally involves the use of beta (electron beam) or gamma radiation emitted by a radioactive isotope (e.g., Cobalt 60 or Caesium 137) to deliver a measurable dose range to the packaged product at room temperature. The radiation is highly penetrative and breaks down chromosomal DNA to inactivate microbes. As ionizing radiation is known to affect polymer properties, the product functionality must be validated at the maximum possible dose administered. Changes in the mechanical, surface, and thermal properties are observed in polyurethanes and depend on the initial polymer chemical structure.^11^ However, Cottam et al. (2009) concluded that gamma radiation causes both chain scission and cross‐linking of a PCL scaffold, yet does not cause significant changes in its properties in the context of tissue engineering.^12^

Alcohol sterilization involves completely submerging the product and soaking in a 70% (v/v) ethanol (or isopropanol [IPA]) solution for a minimum length of time; however, it is not a sterilization method approved by the regulatory bodies. The mode of action is postulated to be rapid protein denaturation^13^ and is effective against viruses, bacteria, and fungi, but not bacterial spores^14^ and hydrophilic viruses,^15^ making this technique insufficient in sterilizing products for medical use. Alcohols are also nonpenetrative (i.e., unlike gamma radiation, they cannot pass through solid materials), and thus cannot ensure terminal sterility. Nevertheless, it is common to sterilize products using ethanol or IPA for research purposes due to their low cost, availability, and ease of use. It should be noted that ethanol has no effect on polymer morphology or molecular weight.^15^

For the electrospun PCL scaffold, gamma irradiation has been shown to negatively affect the surface topography,^16^, ^17^ molecular weight,^11^, ^12^ hydrophobicity,^11^ and mechanical properties.^11^, ^18^ Published work on the effects of ethanol sterilization on electrospun PCL or electrospun polyester scaffolds is less prevalent as it is not a validated sterilization method. However, one study concluded that ethanol sterilization of a poly(lactide‐co‐glycolide) (PLGA) scaffold caused no loss of volume or change in molecular weight of the scaffold.^15^

The aim of this article was to determine the effects of gamma and ethanol sterilization, as well as time, on the functionality of the PCL scaffold after their implantation *in vivo*. Furthermore, implanted scaffolds were manufactured at two separate sites to determine device transfer and scalability in the future.

## METHODS

### Scaffold manufacture and sterilization

Commercially available medical grade PCL (PC12, Purac) with molecular weight 1.2 × 10^5^ g/mol was used by two separate manufacturing sites—by an in‐house manufacturer, University of Manchester, UoM, and by an external manufacturer, The Stellenbosch Nanofiber Company (SNC)—to create electrospun PCL fiber scaffolds.

UoM scaffolds were prepared by electrospinning a 10%w/v solution of PCL dissolved in 1,1,1,3,3,3‐hexafluoroisopropanol (>99% purity, Sigma) targeted toward an earthed thin‐edged mandrel (3 mm width, 600 RPM) positioned at 20 cm from the end of the needle. The polymer solution was expelled from the end of the needle‐tipped syringe at 20 kV (applied voltage) and 1 mL/h (flow‐rate). Collected fibers were removed as a single ribbon and manually twisted into yarns (as described in Ref. 2).

SNC scaffolds were prepared using high‐throughput electrospinning technology—SNC Ball Electrospinning Technology (SNCBEST™)—where partially aligned nanofibers were collected onto a rotating mandrel coated with foil (order number: PCL01yrn). The nanofibers were subsequently rinsed with 70%v/v ethanol (EtOH), followed by deionized water and rinsed again with 70%v/v EtOH before being left to dry at room temperature. SNC nanofibers sheets subsequently received at UoM were cut into thin ribbons (3 × 400 mm), where fiber alignment was parallel to the ribbon length, and manually twisted to create nanofibrous yarns.^2^

Twisted yarns were sterilized either by gamma irradiation (25 kGy, Synergy Health) or by submerging them in increasing concentrations of EtOH (70–100% v/v), 12 h per concentration, followed by rinsing several times in sterile phosphate‐buffered saline (PBS). Ethanol sterilization and PBS washes were performed within a class II microbiological safety cabinet.

### Scaffold implantation into a murine tendon model

**Figure 2:**
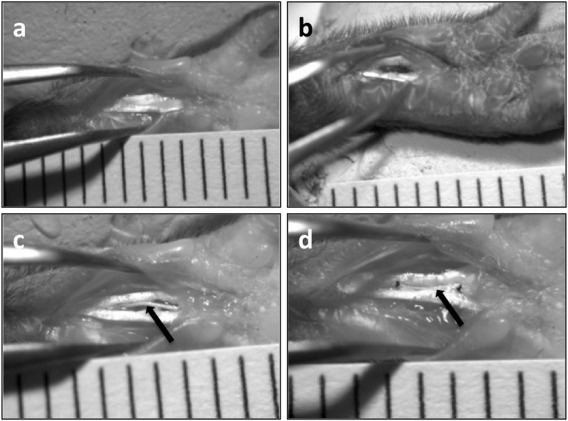
Surgical images depicting the surgical process: (a) location and exposure of the flexor digitorum longus tendon; (b) creation of a critical sized defect; (c) positioning of an electrospun yarn; and (d) single‐knot sutures at either end of the yarn to hold in position. Scale = 1 mm intervals; arrows highlight the yarn location.

The *in vivo* study was conducted over a 6‐week period, with time points at 3 days, 3 weeks, and 6 weeks (Fig. 2). Procedures followed the standards set out by the University of Manchester and in the Animals (Scientific Procedures) Act (1986), UK. Project license 40/3369.

C57/Bl male mice (Envigo, UK) at 8–10 weeks of age were anaesthetized by isoflurane (Abbot Laboratories Ltd, Kent, UK) induction (4 L/min of oxygen with 4% isoflurane). Once induced, mice were maintained at 2 L/min oxygen with 2% isoflurane for the rest of the procedure. A tourniquet was applied securely to mice hind limbs, immobilized in blue tack with the palmar side facing up, and cleaned with an ethanol wipe. Under a Leica MZ7.5 operating microscope (Leica Microsystems, Switzerland), a size‐11 scalpel blade was used to lacerate the epidermal layer of the limb to expose the flexor digitorum longus (FDL) tendon. Using forceps and Vannas Spring Scissors (Fine Science Tools Inc., Germany), a 3 × 1 mm segment of the FDL in the hindpaw of a mouse was resected. Yarns were then implanted and affixed by a single figure‐of‐eight suture (10–0 Dafilon, B. Braun) at either end. Tendon autografts were also performed in the opposite hindpaw using the excised tissue to act as positive controls. The wound was closed and sutured and the mice were left to recover from the surgery in individual cages. At the appropriate time point, mice were euthanized by complete dislocation of the neck and whole hindpaws were harvested for fixation.

### Tissue and scaffold harvesting

Samples were fixed in a zinc fixative for 24–48 h, then processed and sectioned (5 µm thick), and placed on polysine slides and stained with hematoxylin and eosin (H&E). Four representative sections were taken from each sample (*n* = 4) at each time point (*n* = 3). Slides were scanned using a Pannoramic 250 Flash II digital slide scanner and images were saved to allow for analysis on the Pannoramic Viewer software. Additional odd tissue sections (*n* = 2) between the even H&E‐stained slides were stained with eight immunohistochemical markers (Table 1) for each time point. For all experiments, four sample types (Table 1) were analyzed at each of the three time points and compared to the autograft control group.

**Table 1:** Sample Types Implanted and Analyzed at Each Time Point with Corresponding Description and Method of Sterilization, and Antibody Markers Investigated and Their Associated Analytical Parameters

Sample Name	Description	Sterilization Method
Autograft	A nonsterilized graft of tendon tissue taken from the second FDL hindpaw of the same mouse	N/A
UoM Ethanol	Twisted yarn, electrospun at UoM	Ethanol
UoM Gamma	Twisted yarn, electrospun at UoM	Gamma
SNC Ethanol	Twisted yarn, electrospun by SNC	Ethanol
SNC Gamma	Twisted yarn, electrospun by SNC	Gamma
Antibody Marker	Analytical Parameters	High Presence Indicates
CD45	Presence of leukocytes	Inflammatory response
Ly6G	Presence of neutrophils	
F4/80	Presence of macrophages	
Hsp47	Collagen I–V	Extracellular matrix (ECM) production/density
BRDU	DNA synthesis	Cell proliferation
SMA	Actin formation	Myofibroblast formation
TUNEL	DNA fragmentation	Apoptosis
CD31	Angiogenesis, integrin activation	Tissue ingress and growth

### Cell infiltration

**Figure 3:**
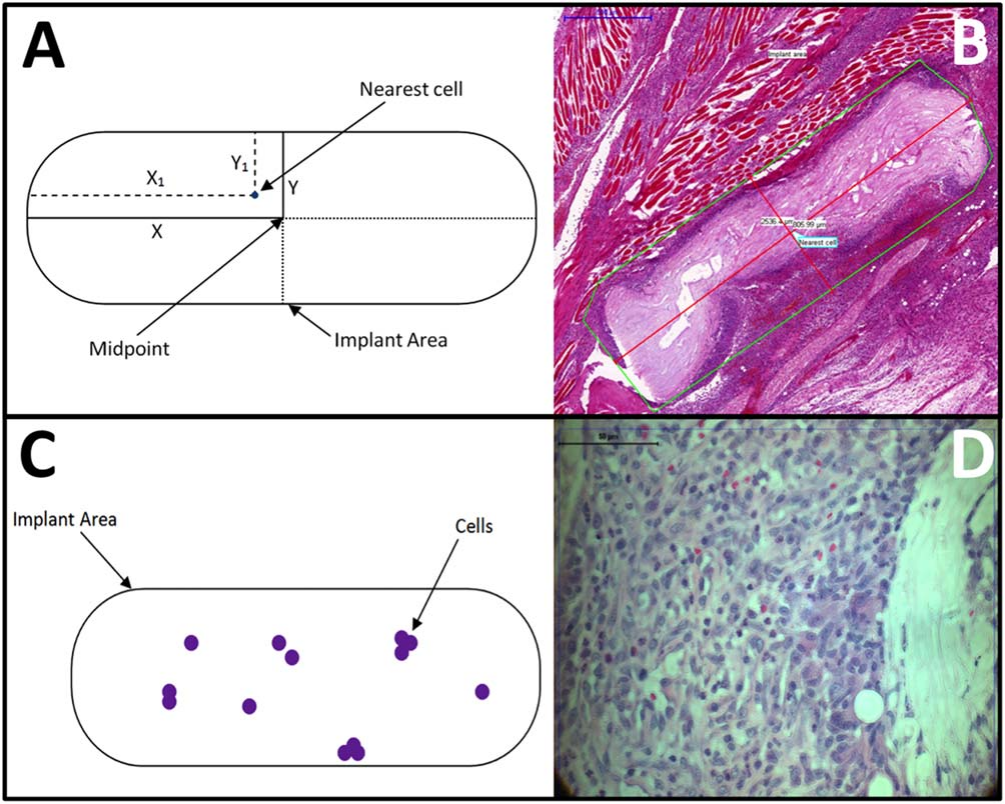
Top image demonstrating schematic of cell infiltration methodology. A: X and Y are half the implant length and width, respectively. X_1_ and Y_1_ designate how far along, and into, the implant that the nearest cell has migrated. B: Micrograph of cell infiltration demonstrating the technique on a sample. The green annotation highlights the implant area. The red annotations indicate the implant length and width, and the nearest cell has been annotated with a light‐blue label. Scale bar = 500 µm. Bottom image demonstrating schematic of cell proliferation methodology. C: Purple represents the implant area covered by cells (size exaggerated for illustration purposes), which would then be captured by the “mask” template file. D: An example of a magnified H&E‐stained sample. Cells are stained purple and are clearly visible against the surrounding tissue. Scale bar = 50 µm.

Cell infiltration into the implant area is an indicator of the efficacy of the scaffold in encouraging cellular attachment and migration. H&E‐stained sections were taken from the middle of the graft/tissue. In each case, the central implant location was determined by the position of the sutures, the greatest area of the graft and examining the cell and tissue orientation. Using the freeform annotation tool within the Pannoramic Viewer software, the implant area was marked and labeled. Using the measurement annotation tool, the area was bisected lengthways by eye and the resulting line (2*X*) was further bisected [Fig. 3(A,B)]. The width of the implant was halved to give a value for *Y*. The cell nearest to the midpoint was marked and using the distance measurement tool, the distance along *X* and *Y* were measured to give *X*_1_ and *Y*_1_, respectively.

Cell infiltration was measured using Eqs. (1) and (2). Migration into and along the implant was denoted d*Y* and d*X*, respectively.

(1) $$\frac{Y-Y_{1}}{Y}\;\times \;100=\%\;migration\;into\;implant=dY$$

(2) $$\frac{X\;-\;X_{1}}{X}\;\times \;100=\%\;migration\;along\;implant=dX$$

### Percentage cell coverage

Cell coverage was analyzed to indicate the percentage of cells present within the implant area over time. Similar to that for cell infiltration, H&E‐stained sections were taken from the central location of the graft/tissue, which was determined by the position of the sutures, the greatest area of the graft and examining the cell and tissue orientation. H&E‐stained samples were analyzed using the HistoQuant module for Pannoramic Viewer. Using an example slide, a “mask” template file was created to automatically select all purple and blue areas (i.e., cells) in the slide and calculate the exact area of these cells [Fig. 3(C,D)]. This template file was then used for all sample slides and the cell coverage within the implant area was determined. The individual area of each cell was summed and divided by the implant area to give coverage as a total cell population percentage [Eq. (3)] (note hematoxylin is non‐cell‐specific). Using this process, a total of 84 results were generated and analyzed for each sample type (*n* = 4) over the time period investigated.

(3) $$\frac{Cell\;Area}{Implant\;Area}\;\times \;100=\;Cell\;Coverage\;\left(\%\right)$$

### Immunohistochemical analysis

Following staining with one of the eight antibody markers (Table 1), tissue samples were subjected to qualitative immunohistochemical analysis under a Leica DMRB microscope by two independent observers in a blinded experiment to assess the level of staining. Assessment was performed at the same time (using same microscope in the same location) to minimize variability with different lighting. This allowed sterilized PCL yarns to be directly compared to the autograft control at the same time point. Samples with greater positive staining relative to the autograft were allocated a score between + and +++ and those with less staining allocated a score between − and −−−. No observable difference between the sample and the autograft was signified by “0.” Results from the immunohistochemical analysis were scored and allocated a value from −3 (−−−) to 3 (+++). The mean scores of the assessors were reported (i.e., − and −− for the same sample would equal −2 or −− when averaged) and recorded.

Two primary areas of interest were analyzed: the implant area (region of interest) and the tissue immediately surrounding the implant. Representative images were saved using Leica QWin analysis software.

### Statistical analysis

Results from all the three experiments were analyzed using a Kruskal–Wallis one‐way analysis of variance (ANOVA) (GraphPad Prism 6 software). Sample types were compared to the corresponding autograft control. Each set of results for a single sample type was also compared over time.

## RESULTS

### Cell infiltration

**Figure 4:**
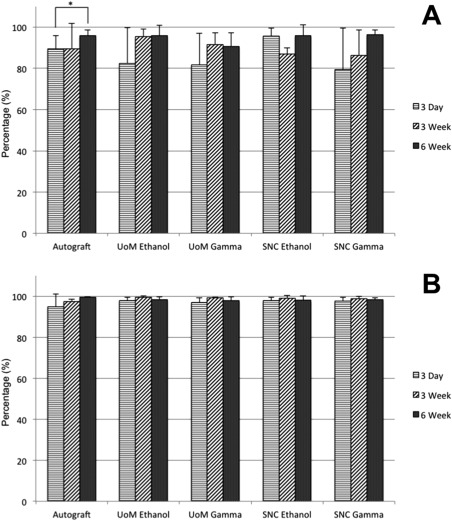
Cell infiltration (%) (A) along the implant (dX) and (B) into the implant (dY) for all scaffolds investigated. A Kruskal–Wallis one‐way ANOVA analysis was applied and highlighted was the significant difference between the autograft samples at 3 days and 6 weeks (*p < 0.05). Data shown as mean and standard deviation (*n* = 4).

Results from cellular infiltration were analyzed and each sample type was compared to the corresponding autograft control at each of the three time points. Infiltration was measured along [(Fig. 4(A)] and into [(Fig. 4(B)] the implant (d*X* and d*Y*, respectively).

Cell migration was at almost 100% in both dimensions for all samples by 6 weeks, signifying cells were able to reach the scaffold's core over the length of the study. Cells in all four scaffold groups and the autograft migrated over 95% of the distance along the implant and over 80% of the distance into the implant within 3 days. Over the study period, migration was slightly greater along the implant compared to movement into the structure. No general trend in mean infiltration over time for any scaffold was observed.

Statistical analysis demonstrated no significant difference between any of the samples and the autograft control for all time points. Similarly, there was no significant difference between any of the time points within each sample set, except for cells infiltrating along the length of the implant (d*X*) for the autograft cohort between 3 days and 6 weeks (*p* < 0.05).

### Percentage cell coverage

**Figure 5:**
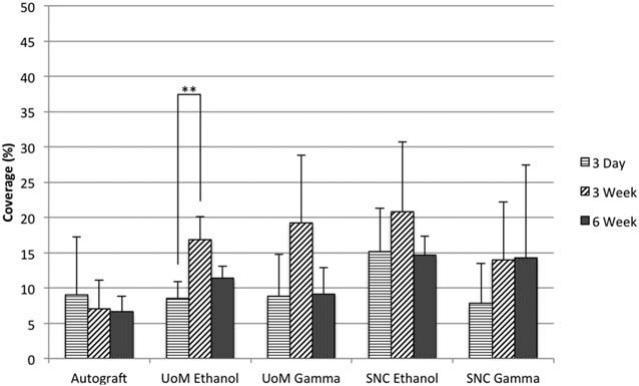
Graph showing cell coverage (%) for all scaffolds. A Kruskal–Wallis one‐way ANOVA analysis was applied and highlighted was the significant difference between the UoM Ethanol samples at 3 days and 3 weeks (**p < 0.01). Data shown as mean and standard deviation (*n* = 4).

Results of cell coverage within the implant were compared to the corresponding autograft control (Fig. 5). Each set of results for a single sample type was also compared over time.

All samples showed above 5% cell coverage in the implant area at each time point, with all sample sets showing a high variance from the mean. Mean coverage in the autograft samples was <10% throughout the study. Only SNC gamma samples indicated a mean coverage that increased with time. SNC Ethanol and both UoM scaffolds had the highest mean coverage at 3 weeks after implantation. No scaffold showed a mean coverage >25%.

Statistical analysis demonstrated no significant difference between any of the samples and the autograft control at any time point. Similarly, there was no significant difference between any of the time points within each sample set, except for between 3‐day and 3‐week results for the UoM Ethanol scaffold (*p* < 0.01).

### Immunohistochemical analysis

**Figure 6:**
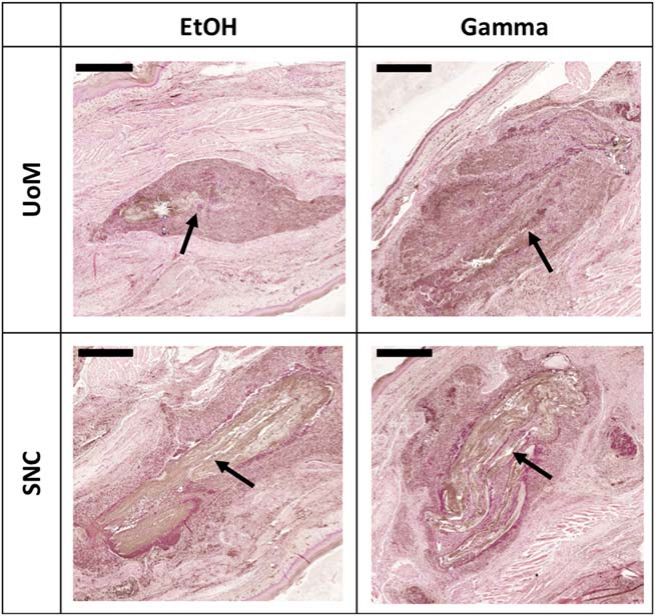
Representative images for one of eight immunohistochemical markers investigated, CD45 (inflammatory marker), at 3 weeks for UoM Ethanol, UoM Gamma, SNC Ethanol, and SNC Gamma. Arrows indicate the implant area. The brown markers (positive staining) are clearly distinguishable against the surrounding pink tissue. Each sample was compared to the autograft. Scale bars = 500 µm.

Immunohistochemical analysis generally showed no difference between scaffold type and corresponding autograft for any of the markers analyzed (Table 2). Results for CD45 demonstrated the most deviation from the autograft. CD45 antibody is an inflammatory marker present in leukocytes^19^ and, consequently, is commonly used as an indicator of inflammation. The results indicate that there is some inflammation in the tissue surrounding all SNC and UoM samples after 3 weeks (representative images Fig. 6). UoM samples sterilized with both gamma and ethanol showed higher density of α‐smooth muscle actin in the area immediately surrounding the implant at 3 weeks.

**Table 2:** Mean Results for Inflammatory Response Markers and Extracellular Matrix and Protein Markers in All Scaffold Types Compared to the Autograft Control

	3 Days		3 Weeks		6 Weeks	
	ROI	SURROUND	ROI	SURROUND	ROI	SURROUND
**A. Inflammatory Response Markers**						
**CD45**						
SNC Ethanol	**++**	**++**	**+**	**++**	**++**	**+**
SNC Gamma	**+**	**++**	**++**	**+**	0	**+**
UoM Ethanol	**+**	**+**	**+**	0	**+**	**+**
UoM Gamma	**++**	**+**	**+**	**+**	**+**	**+**
**F4/80**						
SNC Ethanol	0	0	0	0	**+**	**+**
SNC Gamma	0	0	0	**−**	**+**	0
UoM Ethanol	**+**	**+**	0	**−**	**+**	**+**
UoM Gamma	0	0	**+**	**+**	**+**	**−**
**LY6G**						
SNC Ethanol	0	**+**	**+**	**+**	**++**	**+**
SNC Gamma	**+**	**+**	**+**	0	**+**	0
UoM Ethanol	0	**+**	**+**	0	0	0
UoM Gamma	0	0	**+**	**+**	**+**	0
**B. ECM and Protein Markers**						
**BRDU**						
SNC Ethanol	**+**	0	**−**	**+**	**+**	**+**
SNC Gamma	**+**	0	**+**	**++**	0	**+**
UoM Ethanol	**+**	0	**+**	**+**	**+**	0
UoM Gamma	0	0	**+**	**++**	0	**+**
**HSP47**						
SNC Ethanol	**+**	**+**	0	**+**	**−**	**−**
SNC Gamma	0	0	0	0	**−**	0
UoM Ethanol	0	0	**+**	**+**	0	**+**
UoM Gamma	0	**+**	**+**	**+**	**+**	**+**
**SMA**						
SNC Ethanol	0	0	0	**+**	**−**	**++**
SNC Gamma	**+**	0	0	0	**−**	**+**
UoM Ethanol	0	0	**+**	**+**	**+**	**++**
UoM Gamma	0	0	**+**	**+**	**+**	**++**
**TUNEL**						
SNC Ethanol	0	**+**	0	0	**−**	0
SNC Gamma	**+**	0	0	**−**	**−**	**−**
UoM Ethanol	0	**+**	**+**	**+**	0	0
UoM Gamma	0	0	**+**	**+**	0	0
**CD31**						
SNC Ethanol	**−**	**+**	**−**	**+**	0	0
SNC Gamma	**+**	**+**	0	**+**	**−**	**−**
UoM Ethanol	**+**	**++**	**+**	**+**	0	0
UoM Gamma	0	**+**	**+**	**+**	**−**	0

Both the implant area (region of interest, ROI) and surrounding area (SURROUND) were assessed.

## DISCUSSION

A study into construct cell infiltration was undertaken to determine how far into the implant viable cells were able to migrate to over a 6‐week duration. The presence of viable cells within the implant area after the initial inflammatory stage would indicate if the scaffold was biocompatible and promoted cell infiltration. Our results indicate the presence of cells close to the center of the implant area across all sample types 6 weeks after implantation (Figs. 5 and 6) with a mean infiltration along the implant above 95% at every time point. Thus, the sterilized scaffolds can be deemed to be biocompatible and their structure facilitates infiltration and migration of cells, which is necessary for the long‐term success of the implant. Despite the hydrophobicity of polyesters like PCL,^20^ cells appeared to adhere to the scaffold. This could be due to the rapid deposition of proteins on the scaffold surface following implantation, which provides a hydrophilic surface suitable for cell adhesion.^21^ Cell migration along the implant was generally greater than infiltration into the implant. This could be due to the structure of the implant itself; the twisted yarn structure may need to be optimized to reduce the tightness and increase the lateral porosity of the construct to improve on the cellular infiltration along the fibers and into the implant. The sterilization method and the scaffold manufacturer did not appear to determine the efficacy of cell infiltration at any time point. The significant difference observed between 3‐day and 3‐week autograft samples for migration into the implant could be due to differences in the tissue samples analyzed; the autograft harvested at 3 weeks may simply have possessed a higher number of cells when initially implanted.

Cell coverage was observed to determine what percentage of the implant area was covered with cells over the 6‐week period investigated (Fig. 5). A very high coverage of cells could indicate an inflammatory response, as H&E staining is not cell specific; future studies would aim to elucidate individual cell populations present. Normal tendon tissue has a cell volume of approximately 20%,^22^ but may be higher during the proliferative phase of healing.^23^ The results indicate coverage between 5% and 15% across all sample types including the autograft control over time. There was no significant difference observed between the mean values of each sample type. There was no significant difference in coverage over time for any of the samples, except between 3‐day and 3‐week results for UoM Ethanol. However, there was no significant difference between 3 day and 6 week or 3 week and 6 week for the same sample type. In fact, the percentage cell coverage had decreased by 6 weeks, suggesting that the 3‐week result may be an anomaly. It should be noted that all scaffolds were twisted manually; the UoM scaffold inserted for 3 weeks ethanol may have had a looser structure than other scaffolds, which could have allowed for an increased coverage of cells. The sterilization method and scaffold manufacturer did not appear to affect cell proliferation.

The results also indicate that neither gamma irradiation nor ethanol sterilization had an effect on cellular response to the scaffold. Augustine et al. (2015) concluded that gamma irradiation of electrospun PCL scaffolds led to improved cellular attachment and growth, and that a dose of 35 kGy can sufficiently sterilize the scaffold “without compromising the materials properties” or cell proliferation.^24^ This is in line with the results obtained above, which suggest that gamma irradiation at 25 kGy has no effect on the cellular response to an electrospun PCL scaffold. Bosworth et al. (2012) similarly concluded that gamma irradiation does not affect the cell response of an electrospun PCL scaffold, but only the material properties.^18^ Published results on the effects of ethanol on electrospun polyesters are less common as ethanol is not a recognized method of sterilization. Holy et al. (2001) compared the effects of gamma radiation, ethylene oxide, and ethanol sterilization on a foam PLGA scaffold and concluded that the former two resulted in significant morphological and volume changes compared to ethanol.^15^ The behavior of PLGA is an indicator of how other biodegradable polyesters such as PCL may react to these sterilization techniques. Ethanol did not affect the overall degradation profile. Holy's study demonstrates that ethanol is less degenerative, but the differences between ethanol and gamma sterilization were not observed in this study.

Immunohistochemical analysis of eight antibody markers was undertaken to analyze the extent of implant acceptance, inflammatory response (CD45, Ly6G, and F4/80) and cell proliferation (BrdU), death (TUNEL), and collagen synthesis (Hsp47) into the implant area. Ideally, high levels of matrix deposition, collagen production, cellular proliferation, and the presence of α‐smooth muscle actin (SMA) as a sign of cellular contraction would indicate the onset of tendon assembly. SMA has been identified in tenocytes during critical phases of tendon healing.^25^ It should also be noted that SMA is present during blood vessel formation, which could be indicative of a foreign body response to the implant^26^; however, presence of SMA in a separate 12‐month *in vivo* murine study has demonstrated this not to be indicative of implant encapsulation, and PCL scaffolds positively encouraged collagen deposition compared to autograft controls over this time period.^27^

In general, there was no significant difference observed for any of the sample types at any time point for any of the markers analyzed. The density of CD45 (an inflammatory marker) was slightly higher across all time points for all SNC and UoM samples, except two—SNC 6‐week implant area and UoM 3‐week surrounding area. This indicates elevated levels of inflammation for these samples compared to the autograft control. However, this is expected as synthetic polymers are known to illicit an early inflammatory response as the immune system reacts to the presence of a foreign body.^28^, ^29^ Notably, the manufacturer and sterilization method did not lead to different levels of inflammation.

Overall, no significant difference in cell biological expression markers was observed between any of the scaffolds and the autograft gold standard, suggesting that these scaffolds may offer an alternative intervention for tendon repair.

## CONCLUSION

The sterilized electrospun PCL scaffold functioned similarly to the gold standard autograft control in terms of cell response over a 6‐week time period. Neither ethanol nor gamma sterilization had an observable effect on the functionality of the scaffold when compared to the autograft control. The manufacturer of the electrospun PCL fibers did not have an effect on the performance of the scaffold, which would allow the manufacturing process to be easily outsourced and scaled up for commercial translation. Further long‐term *in vivo* studies are required, but our electrospun PCL scaffold offers promise to becoming an alternative intervention for patients requiring tendon repair.
